# Nanoparticle delivery of combined plant extracts enhances immune response in immunocompromised rats

**DOI:** 10.1038/s41598-025-21329-3

**Published:** 2025-11-07

**Authors:** Selvia S. Milad, Hisham A. Elshoky, Sara E. Ali, Marwa S. Khattab, Mahmoud Z. Attia, Afaf M. Azouz

**Affiliations:** 1 Physiology department, Faculty of Veterinary Medicine, Cairo University, Giza, Egypt; 2 Nanotechnology and Advanced Material Central Lab, Agriculture Research Center, Giza, Egypt; 3 Regional Center for Food and Feed, Agricultural Research Center, Giza, Egypt; 4 Tumor Biology Research Program, Department of Research, Children’s Cancer Hospital, 1 Seket Al-Emam Street, P.O Box 11441, Cairo, 57357 Egypt; 5 Pathology department, Faculty of Veterinary Medicine, Cairo University, Giza, Egypt

**Keywords:** Loaded herbal extracts, Chitosan, Carboxy chitosan nanoparticles, Immunomodulatory, Dexamethasone, Immunosuppressed male rats, Immunology, Physiology

## Abstract

Recently, the world has been dealing with diseases that spread easily and weaken the body’s natural defenses. Boosting natural immunity can help prevent these diseases. This study sought to assess the immunomodulatory effects of extracts from *Illicium verum*, *Saussurea costus*, and *Glycyrrhiza glabra*, both individually and in conjunction with chitosan and carboxy chitosan nanoparticles (CS NPs and C.CS NPs), in immunosuppressed male rats. The primary objective was to assess whether the combined plant extracts exhibit superior immunomodulatory effects compared to single treatments. The herbal extracts were tested chemically. The nanoparticles and loaded herbal extracts were studied for size, charge, and chemical structure, and their safety was tested on two cell types. The study used 104 adult male rats, divided into 13 groups: a control group, an immunosuppressed group, three groups treated with herbal extracts, two nano groups, and six groups treated with loaded herbal extracts. Immunosuppression was caused by injecting 20 mg of dexamethasone weekly. Herbal extracts, CS NPs, C.CS NPs, and the loaded herbal extracts were given orally every day after day for 28 days. The study checked immune health, tissue structure, and cell growth marker Ki-67. All treatments improved immune health by increasing white blood cells, complement 3 and 4, interferon-gamma, and tumor necrosis factor levels, and raised Ki-67 expression. Incorporation of chitosan and methyl carboxy chitosan (C.Cs) nanoparticles amplified the immunostimulatory effect at a reduced dosage of 100 mg/kg, with the C.Cs-based formulation exhibiting enhanced therapeutic performance. The results highlight the promise of nanoparticle-formulated herbal extracts, especially C.CS-loaded variants, as potent immunomodulatory agents with enhanced therapeutic efficacy.

## Introduction

 The primary functions of the immune system are identifying and eliminating foreign entities. Any immune disorders affect negatively on general health, so boosting the immune system assits in controlling dangerous infections and autoimmune conditions^1^. Immunosuppression is a decrease in the immune system’s efficacy, causing an increase in the risk of infections, cancer, and autoimmune disorders. This is caused by factors including aging, illnesses, and medications^2^. Dexamethasone a synthetic corticosteroid medication, is among the immunosuppressive drugs that can cause immunosuppression by binding to glucocorticoid receptors on immune cells like T and B cells, inhibiting their activation and proliferation^3^.

Herbal medicines have been used globally to treat various diseases^4^. Recent approaches have been developed to study the immunostimulatory activity of herbs and their derivatives^5^. Natural extracts are often used as supplementary food in the form of tablets, capsules, granules, and syrups to facilitate the administration of bioactive ingredients^6^. Plant extracts contain a variety of bioactive compounds, including alkaloids, amino acids, enzymes, flavonoids, polyphenols, proteins, reducing sugars, and other biologically active substances^7^.

The study focused on three herb species, *Illicium verum*, *Saussurea costus*, and *Glycyrrhiza glabra*. *Illicium verum* (Chinese star anise) is commonly used in traditional Chinese medicine due to its various bioactive components, such as flavonoids, phenylpropanoids, monoterpenoids, lignans, sesquiterpenoids, and volatile substances. It possesses several therapeutic properties, such as antioxidants, anti-inflammatory, analgesic, antibacterial, antifungal, insecticidal, anticonvulsive, sedative, and anticancer effects^8^. *Saussurea costus* is the most common species, and its active components include terpenes, flavonoids, anthraquinones, alkaloids, tannins, and inulin. The herb’s primary constituents are sesquiterpene lactones, which have demonstrated a range of pharmacologic actions, including anti-inflammatory, antiulcer, anticancer, and hepatoprotective activity^9^. *Glycyrrhiza glabra* (licorice), the Greek words glykos (sweet) and rhiza (root), its extracts are used in the food and pharmaceutical industries. It has been historically utilized to treat various ailments, such as gastric ulcers, tuberculosis, liver disorders, rheumatoid arthritis, Addison’s disease, sore throats, and acts as a laxative, anti-tussive, and expectorant^10^.

Recent advances concerned the potential of nanoparticles in all areas of life sciences. They are useful in biopharmaceuticals, cosmetics, electronics, textiles, medicine, and other industries due to their distinct properties, which also cause various immunological reactions in the body. Nanoparticles provide the potential for improving adjuvants, cytokines, vaccines, and medicines^11^. Moreover, nanoparticles have been widely applied in the medical field for purposes such as disease prevention, diagnosis, and treatment. They are also utilized in medical sensors, imaging techniques, and drug delivery systems^12^.

Nanotechnology-based systems have been used to enhance the properties of herbal extracts, and herbal extract-loaded nanoparticles offer advantages such as size and distinctive physicochemical properties. These can reduce toxicity, provide targeted drug delivery, and improve stability^13^. Chitosan is a byproduct of chitin^14^, has potential in biomedical materials and tissue engineering but is insoluble at neutral or high pH levels^15^. Carboxy chitosan (C.CS), a chitosan derivative, is water-soluble, biocompatible, and has been investigated for use in biomedical materials such as drug delivery carriers, tissue engineering scaffolds, and hemostasis materials^16^.

Therefore, the present study aimed to investigate the immunostimulatory potential of Illicium verum, Saussurea costus, and Glycyrrhiza glabra (Iv, Sc, and Gg) extracts, either individually or in combination with chitosan (CS) and carboxymethyl chitosan (C.CS) nanoparticles, in a dexamethasone-induced immunosuppression model in male rats. The primary objective was to determine whether co-administration of the plant extracts yielded enhanced immunomodulatory effects compared to single treatments and to evaluate the differential efficacy of CS and C.CS nanoparticles as delivery platforms for herbal bioactives.

## Materials and methods

### Nanoparticles preparation

Chitosan nanoparticles (CS NPs) and Carboxy chitosan nanoparticles (C.CS NPs) were produced in the Agricultural Research Center’s Central Laboratory for Nanotechnology and Advanced Materials in Giza, Egypt, using the ionic gelation method^17^. First, 200 mg of low MW chitosan (112000) from Across Organics, USA, or carboxy chitosan (prepared using the method described in^18^) were dissolved separately in 4 ml of glacial acetic acid (MERK, 99%) and 185 ml of distilled water. These mixtures were stirred at room temperature overnight. The nanoparticles were then formed by adding 66 mg of sodium tripolyphosphate and shaking the solution for 30 min.

#### Characterization of nanoparticles

CS and C.CS NPs were characterized using a UV–Vis-NIR spectrophotometer (Cary 5000 UV–Vis-NIR; Varian, U.K.). The zeta potential and size of the NPs were determined using a Zetasizer nano series (Malvern, U.K). Fourier-transform infrared spectroscopy (FTIR) analysis was conducted using a JASCO FT-IR 6100 spectrometer (Japan). Peaks and bands were identified using IRPAL 2.0 (Table - driven Infrared Application) software. The characterization procedures were performed at the Nanotechnology and Advanced Materials Central Laboratory.

### Herbal extract preparation

Three herbs, *Illicium Verum* (fruit), *Saussurea Costus* (root), and *Glycyrrhiza Glabra* (root), were obtained from the regional market (Haraz, Cairo, Egypt). These herbs were identified by the botany department at Cairo University’s Faculty of Science. Afterward, they were washed with distilled water, dried for 10 h in a vacuum oven at 50 $\:^\circ\:\complement\:$, crushed, and ground into a fine powder.

Ethanolic extracts were prepared using the technique described in^19^. The resulting filtrate was collected and concentrated using a rotary evaporator (UK’s IkA RV 10 basic) at 60 °C and 180 rpm to remove the solvent. The extracts were subsequently lyophilized to assess the total solids (extract yield%), flavonoids, phenolic content, and total antioxidant activity. The extraction yield was determined as follows:

Extract yield%= 

$$\:\frac{\text{w}\text{e}\text{i}\text{g}\text{h}\text{t}\:\text{o}\text{f}\:\mathbf{l}\mathbf{y}\mathbf{o}\mathbf{p}\mathbf{h}\mathbf{i}\mathbf{l}\mathbf{i}\mathbf{z}\mathbf{e}\mathbf{d}\:\:\text{e}\text{x}\text{t}\text{r}\text{a}\text{c}\text{t}\:\text{p}\text{o}\text{w}\text{d}\text{e}\text{r}}{\text{I}\text{n}\text{i}\text{t}\text{i}\text{a}\text{l}\:\text{w}\text{e}\text{i}\text{g}\text{h}\text{t}\:\text{o}\text{f}\:\text{h}\text{e}\text{r}\text{b}\:\text{p}\text{o}\text{w}\text{d}\text{e}\text{r}}\:\times\:100$$

#### Evaluation of total antioxidant activity

Antioxidant activity was assessed using the 2, 2’-Diphenyl-1-picrylhydrazyl (DPPH) method^20^. The percentage inhibition of the DPPH radical was calculated using the following formula:

Antioxidant activity% = 

$$\:\frac{\text{A}\text{c}\:\left(0\right)\:-\:\text{A}\text{A}\:\left(\text{t}\right))\:}{\text{A}\text{c}\:\left(0\right)}\times\:\:100$$

Where: Ac (0) is the absorbance of the DPPH reagent without a sample (measured at 0 min), and AA is the absorbance of the antioxidant after 1 h.

#### Evaluation of phenolic content

Phenolic content was evaluated using the method described by^21^ with modifications. 1 ml of the extract solution was blended with Folin-Ciocalteu reagent (5 ml, previously diluted with water 1:10 v/v) and 4 ml of sodium carbonate (75 g/l). The tubes were vortexed for 15 s, and color development was allowed to occur during a 30-minute incubation period at 40 $\:^\circ\:\complement\:$. Absorbance was measured at 765 nm. The standard curve was calculated using gallic acid, and the results were given as mg of gallic acid equivalents (GAEs) per g of extract.

#### Evaluation of flavonoid content

The method given by^22^ was modified to determine the flavonoid content. A portion of the extract solution (0.5 ml) was mixed with distilled water (2 ml), then with 5% NaNO_2_ solution (0.15 ml). AlCl_3_ solution (10%, 0.15 ml) was added after 6 min, and the mixture was then given another 6 min to stand. 2 ml of NaOH solution (4%) was added to the mixture. The solution was thoroughly mixed, and distilled water was added to make the final volume 5 ml. It was then let stand for 15 min. At 510 nm, the intensity of pink was quantified. Using (+)-catechin to create the standard curve, the results were represented as mg of (+)-catechin equivalents (CEs) per g of extract.

### Preparation of loaded herbal extracts

To prepare the loaded herbal extracts, 25 ml of each herbal extract was mixed with 25 ml of CS NPs to form CS-loaded herbal extracts, or with 25 ml of C.CS NPs to form C.CS-loaded herbal extracts, both in a 1:1 ratio. These mixtures were shaken overnight. On the following day, 0.5 ml of 1% polyethylene glycol (PEG 8000) was added dropwise to the solution and shaken for 2 h^23^.

#### Loading efficiency

To evaluate the loading efficiency of nanoparticles loaded with herbal extracts, The pH of nanoparticles loaded herbal extracts solution is raised to 9 using NaOH to enhance the solubility and stability of the herbal extracts. The pellets were then dissolved in 1% glacial acetic acid (MERK, 99%), and the solution was centrifuged at 5000 rpm for 45 min to separate the unencapsulated extracts from the loaded nanoparticles. The following formula was used to determine loading efficiency:

Loading efficiency % **=**^24^

$$\:\frac{\text{a}\text{b}\text{s}\text{o}\text{r}\text{b}\text{a}\text{n}\text{c}\text{e}\:\text{o}\text{f}\:\text{p}\text{e}\text{l}\text{l}\text{e}\text{t}\:}{\text{a}\text{b}\text{s}\text{o}\text{r}\text{b}\text{a}\text{n}\text{c}\text{e}\:\text{o}\text{f}\:\text{p}\text{e}\text{l}\text{l}\text{e}\text{t}+\text{a}\text{b}\text{s}\text{o}\text{r}\text{b}\text{a}\text{n}\text{c}\text{e}\:\text{o}\text{f}\:\text{s}\text{u}\text{p}\text{e}\text{r}\text{n}\text{a}\text{t}\text{a}\text{n}\text{t}\:}\:\times\:100$$

#### Energy dispersive X-Ray fluorescence (EDXRF)

A non-destructive analysis technique known as Energy Dispersive X-Ray Fluorescence (EDXRF) was utilized at Chemical Ware Fare’s Main Laboratories in Cairo, Egypt. The purpose was to examine the elemental composition of samples without causing any harm. The analysis was performed using the Thermo Scientific ARL OPTIM’X Fuel & Lubricant Analyzer.

### Cytotoxicity evaluation

To evaluate cytotoxicity, human lung fibroblast (WI-38) and embryonic kidney (HEK-293) cells were used. The Nile Center (Mansoura, Egypt) provided the WI-38 cells, while Dr. Salma Tammam provided the HEK-293 cells (GUC University, Cairo, Egypt). In 96-well plates, cells were seeded at a density of 1 × 10^4^ cells per well in 200 µl of growth medium (Dulbecco’s modified Eagle media containing 10% fetal bovine serum) and incubated for an overnight period at 37$\:^\circ\:\complement\:$ $\:.$ The growth medium was then swapped out for 200 µl of new growth medium containing 0, 1, 4, 10, 20, and 40 ng/ml dexamethasone as well as additional materials (CS NPs, C.CS NPs, as well as Iv, Iv-CS, Iv-C.CS, Sc, Sc-CS, Sc-C.CS, Gg, Gg-CS, and Gg-C.CS) at concentrations of 0, 100, 500, 1250, 2500, and 5000 ng/ml. The medium was changed after 24 h with 100 µl of new medium.

Cell viability (%) =^25^

$$\:\:\frac{Sample\:absorbance}{Control\:absorbance}\times\:100\:\:\:\:\:\:\:\:\:\:\:\:\:\:$$

### Animals

One hundred and four mature male albino Sprague-Dawley rats, each weighing 200 ± 5 g, were received from the Faculty of Veterinary Medicine, Cairo University. They were reared at the physiology department in plastic cages with wood shaving bedding in a well-ventilated environment at 22 ± 3 $\:^\circ\:\complement\:$ and 55 ± 5% humidity under a 12/12 h light- dark cycle. Rats were given regular lab chews and free access to water.

#### Experimental design

**Fig. 1:**
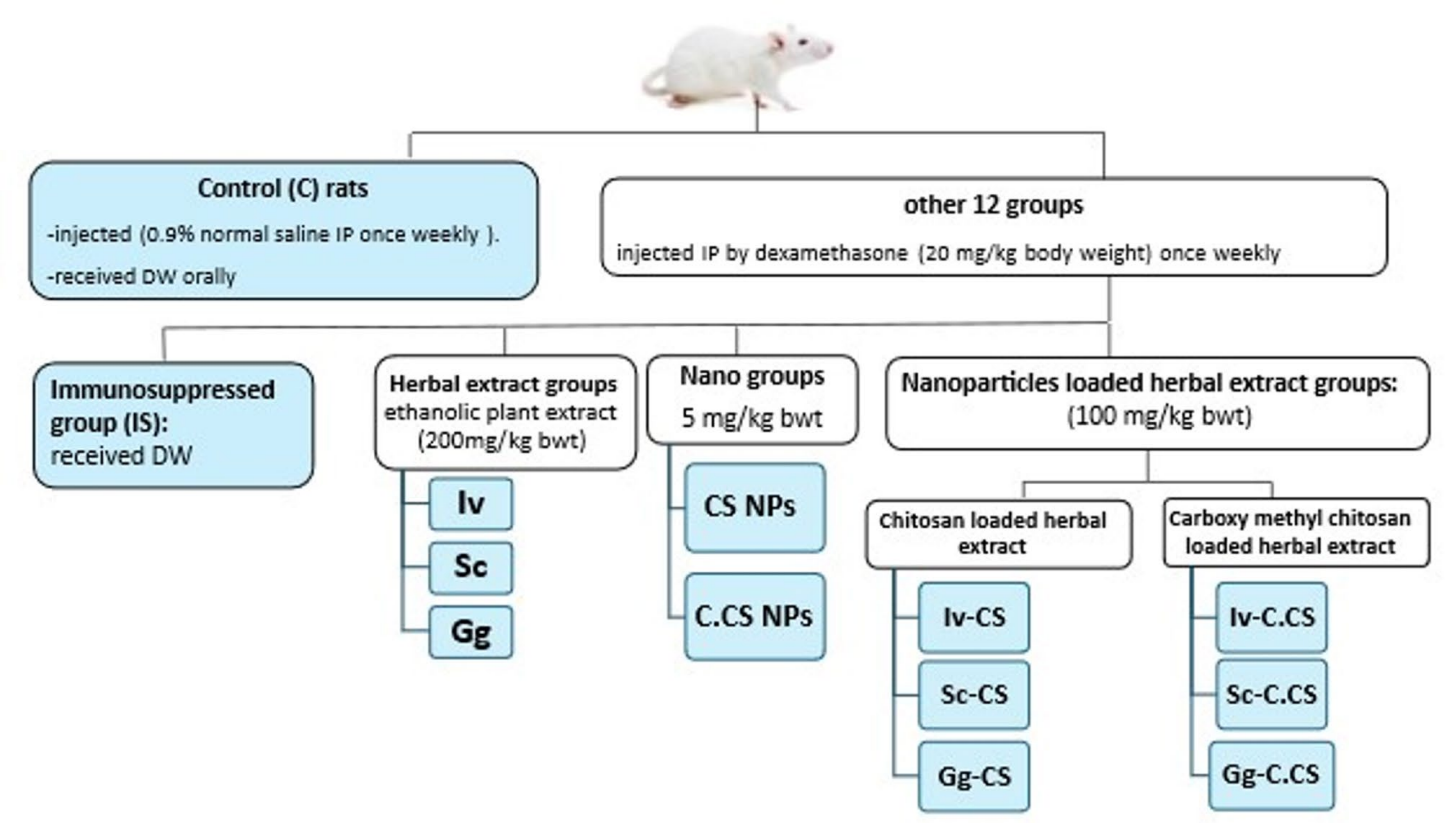
The experimental grouping of rats.

The Institutional Animal Care and Use Committee of the Veterinary Medicine, Cairo University accepted the experimental protocol (Vet CU 2305 2022449). After two weeks of acclimatization, the rats were divided randomly into thirteen groups, eight of each. The experimental period lasted for 28 days as shown in Fig.1. Immunosuppression was induced by IP injection of dexamethasone (20 mg/kg body weight) once weekly^23^. Herbal extracts were received orally (200 mg/kg bwt day after day)^26–28^. Nanoparticles were received orally (5 mg/kg bwt day after day)^29^. Nanoparticles loaded herbal extracts were received at 100 mg/kg bwt day after day^30^.

Rats were anesthetized with 91 mg/kg ketamine injected intraperitoneally at days 14 and 28. Blood was drawn into two different tubes, EDTA tubes to obtain anticoagulated whole blood samples for total leucocytic counts and gel separator tubes centrifuged at 3000 rpm for 10 min at 4 °C to obtain serum for immunological analysis, liver, and kidney function. All rats were sacrificed by cervical dislocation after blood collection at day 28.

### Immunological status evaluation

Total leucocytic count (TLC) was manually determined in anticoagulated blood using a light microscope^31^.Other immunological parameters such as interferon gamma (IFN) (E-EL-R0009) complement 3 (C3) (E-EL-R0250), complement 4 (C4) (E-EL-R0248), and tumor necrosis factor (TNF) (E-EL-R0019), were evaluated in serum using rat-specific enzyme-linked immunosorbent assay kits (Elabscience company, Egypt) following the manufacturer’s guidance.

### Biochemical parameters analysis

The liver enzymes, Alanine aminotransferase (ALT) and Aspartate aminotransferase (AST) activities were evaluated following the technique^32^ using colorimetric kits (2.6.1.2, 2.6.1.1, respectively, Diamond, Egypt).

Total protein (Tp) and albumin (Alb) levels were measured following the methods described in^33,34^, respectively, using kits (catalog numbers 310001 and 211001, Spectrum, Egypt, respectively). Globulin (Glo) levels were calculated as follows: $\:\text{G}\text{l}\text{o}\:\text{l}\text{e}\text{v}\text{e}\text{l}\text{s}\text{\:=\:total\:protein\:levels\:-\:albumin\:levels\:}$, disregarding fibrinogen amounts as mentioned by^35^. The Alb/Glo ratio was also calculated.

The kidney function was evaluated by estimating urea and creatinine levels according to the method of^36^ using commercial kits (Diamond- Egypt for urea and Spectrum -Egypt for creatinine: 234001).

### Histopathological examination

Tissue samples were collected from the spleen, liver, and kidney and placed in 10% neutral buffered formalin for fixation. Tissues were then processed by the paraffin embedding technique. A rotary microtome (Leica 2135, Germany) was used to cut 4 μm - thick tissue sections from the paraffin blocks. Tissue sections were then stained by hematoxylin and eosin stain and examined by a light microscope (BX50F4, Olympus, Japan) equipped with a digital camera for photography of tissue.

### Immunohistochemistry of Ki-67

Paraffin - embedded tissue sections were deparaffinized, rehydrated, and then placed in sodium citrate buffer, PH 6, for antigen retrieval. Primary polyclonal Ki-67 antibody (PA5-19462) was applied to slides and incubated overnight. Hydrogen peroxidase was added to slides for 10 min to eliminate the endogenous peroxidase. Secondary biotinylated antibody and the avidin peroxidase complex (Vac-tastain ABC peroxidase kit, Vector Laboratories, Burl-ingame, CA, USA) were then added to slides. 3, 3-diaminobenzidine was used for color development (DAB, Sigma Chemicals, Perth, Australia). Phosphate buffer saline was used for washing twice for 5 min between every step. The area percent of positive brown staining was measured by Image J software in 5 photos/rat at 400X magnification.

### Statistical analysis

The results were analyzed using a one-way analysis of variance (ANOVA) and Duncan post-hoc test, utilizing SPSS software (Version 20) to identify group differences. Data are reported as means ± standard error. Significant differences are indicated by different lowercase letters for day 14 (*P* ≤ 0.05) and uppercase letters for day 28 (*P* ≤ 0.05). Additionally, a paired sample t-test was performed to compare results within the same group across days 14 and 28. Significant differences are marked by symbols: * for *P* ≤ 0.033, ** for *P* ≤ 0.002, and *** for *P* ≤ 0.001.

## Results

### Characterization of nanoparticles

**Fig. 2:**
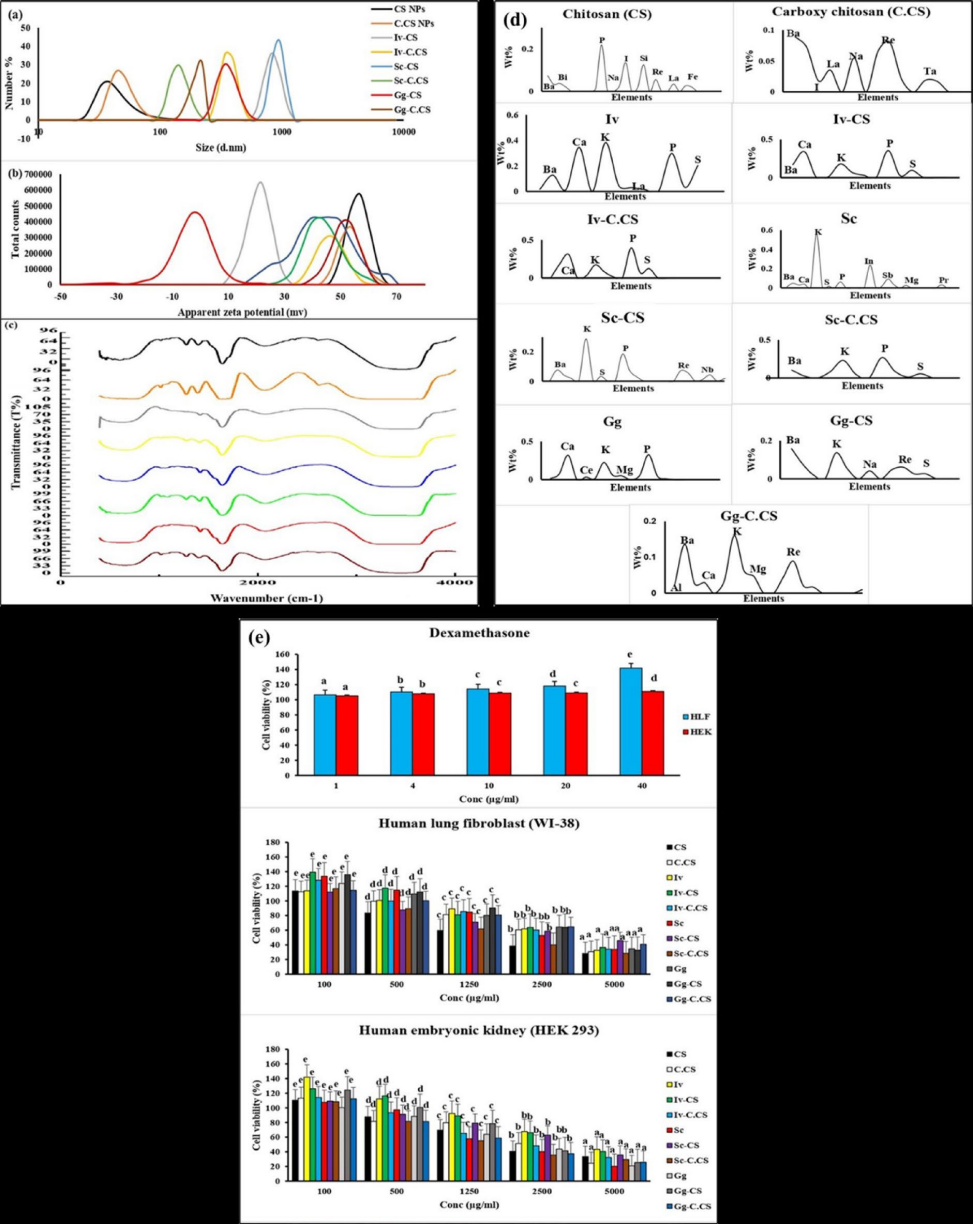
(**a**-**c**) Characterization of nanoparticles (CS NPs, andC.CS NPS), and loaded herbal extracts (Iv-CS, Iv-C.CS, Sc-CS, Sc-C.CS, Gg-CS and Gg-C.CS) (), ()) (**a**) particles size (**b**) zeta potential, and (**c**) FTIR. (**d**) Energy dispersive x-ray fluorescence of herbal extracts (Iv, Sc, Gg), nanoparticles, and loaded herbs. (**e**) The cytotoxicity profiles of dexamethasone, CS and, C.CS nanoparticles, herbal extracts, and herbal extract-loaded formulations were evaluated using human lung fibroblast (WI-38) and human embryonic kidney (HEK-293) cells through the WST-1 assay. Results are expressed as mean ± Sc (*n* = 3) and analyzed via one-way ANOVA followed by Duncan’s multiple range test. Significant differences (*p* < 0.05) are indicated by different letters.

A common technique for determining particle size and particle size distribution (PSD) is dynamic light scattering (DLS)^37^. The size of CS NPs and C.CS NPs was about 46.21 nm ± 28.75 nm and 51.72 ± 24.74 nm, respectively. C.CS loaded herbs are characterized by a larger size. Size of Iv-CS, Iv-C.CS, Sc-CS, Sc-C.CS, Gg-CS, Gg-C.CS were about 844 ± 128.4 nm, 368.9 ± 54.09 nm, 924 nm ± 112.6 nm, 145 ± 24.88 nm, 354.2 ± 171.8 nm, 361.8 ± 68.38 nm, 354 ± 171.8 nm, respectively (Fig. 2a). Due to the presence of solid material in the extract and the high concentration of the utilized extracts, the size of the C.CS - loaded herbs has increased dramatically.

The electrophoretic light scattering (ELS) technique is used to determine the zeta potentials of NPs to assess their stability^38^. Therefore, the efficiency of NPs and their interactions with target molecules can be influenced by their zeta potentials. The zeta potential measurements were 62.6 ± 5.90, 53.6 ± 4.24, 22,4 ± 4.34, 32.6 ± 4.64, 39.4 ± 7.04, 30.6 ± 6.23, 34.5 ± 8.70, 36.9 ± 4.55 mV for CS NPs, C.CS NPs, Iv-CS, Iv-C.CS, Sc-CS, Sc-C.CS, Gg-CS, and Gg-C.CS respectively (Fig. 2b).

FTIR is a crucial tool to distinguish between CS NPs, C.CS NPs, Iv-CS, Iv-C.CS, Sc-CS Sc-C.CS, Gg-CS, and Gg-C.CS respectively. It displays various FTIR spectra between 400 and 4000 cm^−1^. Figure 1c. showed the difference between Cs and C.CS NPs due to the chemical modification in Cs NPs. Bands at approximately 1018, 1280, 1288, 1558, 1396, and 3263 cm-1 were assigned to carboxylic acid. The band at 1419 was appointed to aromatics. Bands at 1635 and 3502 cm^−1^ were dedicated to amides. The band at 1666 was designated to alkenes, but band at 1118, 1149 and 3448 cm-1, which were assigned to amines. Appearance of band at 1666 cm^−1^ due to C-C stretching. The bands at 1018, 1396, 1635, 2615 shifted to 1010,1388, 1620, 2623 cm-1 respectively in C.CS NPs due to C-O stretching. According to the appearance and shift bands, this confirms bonding between the carboxy methyl group and the chitosan carbon backbone and successful modification of Chitosan. In all, C.CS loaded herbs band at 1010 to 1018 cm^−1^ due to C-O stretching. Appearance of band at 1087 cm^−1^ in Iv-C.CS and Sc-C.CS due to C-O stretching. Additionally, bands at 1273 shifted to 1149 cm^−1^ in Iv-C.CS, 1388 shifted to 1396 cm^−1^ in Gg-C.CS due to C-O stretching. Moreover, bands at 1388 shifted to 1411 cm^−1^ in Sc-C.CS and Gg-C.CS due to S = O ester group. Absorption of band at 1666 cm^−1^ in all C.CS loaded with herbal extracts due to C = N stretching.

In CS loaded herbs the appearance of a band at 1118 cm-1 in Sc-CS is only due to C-O stretching, bands at 1118 cm-1 in Iv-CS and 1149 cm-1 in Sc-CS, and Gg-CS due to C-N stretch. Moreover, the appearance of bands at 1280 cm-1 in Sc-CS and 1288 cm-1 in Iv-CS, Gg-CS is due to C-O stretching. Appearance of band at 1419 cm-1 in all CS loaded herbs due to Ar C-C stretching. Bands at 1558 cm-1 due to C-O stretching. Additionally, the appearance of bands at 1635 cm-1 in all CS loaded herbs is due to C-O stretching (Fig. 2c).

### Phytochemical analysis

The DPPH free radical scavenging method was employed to determine the antioxidant capabilities of herbal extracts in vitro. Among the extracts, Iv exhibited the highest antioxidant activity (93.74%), whereas Gg and Sc demonstrated lower activity, with values of 87.92% and 82.83%, respectively. Furthermore, the loaded illicium extracts (Iv-CS and Iv-C.CS) displayed higher antioxidant activity (80.33% and 84.13%, respectively) compared to glycyrrhiza (Gg-CS: 68.77%, Gg-C.CS: 73.20%) and saussurea (Sc-CS: 65.37%, Sc-C.CS: 70.69%). Overall, all C.CS loaded herbal extracts demonstrated higher phenolic and flavonoid content, as well as antioxidant activity, compared to CS alone.

The Folin - Cicalteau method was used to analyze the phenolic content in both loaded and unloaded herbal extracts due to the importance of phenolic compounds and their antioxidant properties. The results presented in Table 1 showed considerable variations in the phenolic and flavonoid contents among the samples. Iv had the highest content of phenolic and flavonoid (23.04 mg GAE/g and 19.76 mg QE/g, respectively), followed by Gg (19.03 mg GAE/g and 14.07 mg QE/g), while the lowest values were observed in Sc (14.74 mg GAE/g and 11.93 mg QE/g). Additionally, loaded illicium extracts displayed higher phenolic and flavonoid content (2.18 mg GAE/g, 2.41 mg QE/g for Iv-CS and 2.43 mg GAE/g, 2.70 mg QE/g for Iv-C.CS) compared to glycyrrhiza (2.02 mg GAE/g, 1.49 mg QE/g for Gg-CS and 2.29 mg GAE/g, 1.81 mg QE/g for Gg-C.CS) and saussurea (1.55 mg GAE/g, 1.33 mg QE/g for Sc-CS and 1.96 mg GAE/g, 1.44 mg QE/g for Sc-C.CS). Finally, the chitosan nanoparticles exhibited the lowest phytochemical contents compared to C.CS, loaded and unloaded herbal extracts.

**Table 1:** Analysis of phenolics and flavonoids and antioxidant activity of herbal extracts, loaded herbs, and nanoparticles.

Material	Total antioxidant (%)	Phenolics (mg of gallic acid equivalents (GAEs)/g of extract)	Flavonoids (mg of chatequin equivalents (CEs)/g of extract)
**CS**	16.70 ^**a**^±0.06	0.13 ^**a**^ ±0.0004	0.33 ^**a**^±0.002
**C.CS**	22.66 ^**b**^±0.08	0.14 ^**a**^±0.0008	0.45 ^**b**^±0.002
**Iv**	93.74 ^**k**^±0.39	23.04 ^**j**^±0.008	19.76 ^**k**^±0.03
**Iv-CS**	80.33 ^**g**^±0.09	2.18 ^**e**^±0.013	2.41 ^**g**^±0.007
**Iv-C.CS**	84.13 ^**i**^±0.1	2.43 ^**g**^±0.01	2.70 ^**h**^±0.006
**Sc**	82.83^**h**^ ± 0.41	14.74 ^**h**^±0.012	11.93 ^**i**^±0.03
**Sc-CS**	65.37 ^**c**^±0.05	1.55 ^**b**^±0.0009	1.33 ^**c**^±0.005
**Sc-C.CS**	70.69 ^**e**^ ±0.11	1.96 ^**c**^±0.0008	1.44 ^**d**^±0.005
**Gg**	87.92 ^**j**^±0.12	19.03 ^**i**^±0.009	14.07 ^**j**^±0.03513
**Gg-CS**	68.77 ^**d**^±0.08	2.02 ^**d**^±0.001	1.49 ^**e**^± 0.006
**Gg-C.CS**	73.20 ^**f**^±0.05	2.29 ^**f**^±0.007	1.81 ^**f**^±0.005

Values have the different superscripts are significantly different at *P* ≤ 0.05 according to one-way ANOVA followed by the Duncan test

### Loading efficiency

The loading efficiency of loaded herbal extracts was calculated according to the following formula: Loading efficiency% **=**

$$\:\frac{\text{a}\text{b}\text{s}\text{o}\text{r}\text{b}\text{a}\text{n}\text{c}\text{e}\:\text{o}\text{f}\:\text{p}\text{e}\text{l}\text{l}\text{e}\text{t}\:}{(\text{a}\text{b}\text{s}\text{o}\text{r}\text{b}\text{a}\text{n}\text{c}\text{e}\:\text{o}\text{f}\:\text{p}\text{e}\text{l}\text{l}\text{e}\text{t}+\text{a}\text{b}\text{s}\text{o}\text{r}\text{b}\text{a}\text{n}\text{c}\text{e}\:\text{o}\text{f}\:\text{s}\text{u}\text{p}\text{e}\text{r}\text{n}\text{a}\text{t}\text{a}\text{n}\text{t})\:}\:\times\:100$$

Loading efficiency was 49.85%, 50.44%, 50.09%, 50.07%, 50.34%, 49.60% for Iv-CS, Iv-C.CS, Sc-CS, Sc-C.CS, Gg-CS, and Gg-C.CS respectively.

### Energy dispersive X-Ray fluorescence analyses (EDXRF)

Nanoparticles (CS NPs, and C.CS NPs,), herbal extracts (Iv, Sc, and Gg), herbal extract-loaded CS NPs (Iv-CS, Sc-CS, and Gg-CS), and herbal extract-loaded C.CS NPs (Iv-C.CS, Sc-C.CS, and Gg-C.CS) are composed of a variety of minerals in differing amounts, CS consists of barium (0.0333%), bismuth (0.0376%), sodium (0.0273%), iodine (0.136%), silicon (0.125%), rhenium (0.0554%), lanthanum (0.0341%), and iron (0.0263%), C.CS comprises barium (0.0895%), bismuth (0.0721%), iodine (0.0176%), lanthanum (0.0352%), sodium (0.0548%), phosphorus (0.0652%), rhenium (0.0805%), and silicon (0.0205%).

Iv comprises Aluminum (0.014%), barium (0.127%), bismuth (0.0138%), calcium (0.345%), potassium (0.383%), lanthanum (0.0456%), magnesium (0.0298%), manganese (0.0142%), phosphorus (0.297%), rhenium (0.0327%), and Sulfur (0.206%). Iv-CS consists of barium (0.168%), calcium (0.341%), iodine (0.0123%), potassium (0.18%), lanthanum (0.0681%), magnesium (0.0294%), phosphorus (0.356%), rhenium (0.0272%), and sulfur (0.0953%). Iv-C.CS comprises barium (0.182%), calcium (0.309%), potassium (0.173%), lanthanum (0.0803%), magnesium (0.0155%), phosphorus (0.395%), rhenium (0.0694%), and sulfur (0.124%).

Sc consists of barium (0.0481%), calcium (0.0385%), potassium (0.564%), sulfur (0.0178%), phosphorus (0.0624%), indium (0.239%), antimony (0.0922%), praseodymium (0.0305%), and magnesium (0.0245%). Sc-CS is made up of barium (0.075%), potassium (0.288%), sulfur (0.0332%), phosphorus (0.185%), indium (0.0904%), rhenium (0.0712%), niobium (0.0426%), and zirconium (0.0184%). Sc-C.CS includes barium (0.105%), calcium (0.0257%), iodine (0.0825%), potassium (0.231%), lanthanum (0.0459%), phosphorus (0.268%), rhenium (0.0887%), silicon (0.0122%), and sulfur (0.0548%). Gg contains aluminum (0.0118%) barium (0.0725%), calcium (0.319%), iodine (0.0135%), potassium (0.223%), lanthanum (0.0568%), magnesium (0.0493%), sodium (0.0537%), phosphorus (0.327%), rhenium (0.043%), and silicon (0.0102%). Gg-CS contains barium (0.16%), bismuth (0.0782%), calcium (0.0195%), potassium (0.136%), lanthanum (0.0533%), sodium (0.0416%), phosphorus (0.0483%), rhenium (0.0609%), silicon (0.026%), and sulfur (0.0265%). Finally, Gg-C.CS contains aluminum (0.0256%), barium (0.136%), bismuth (0.0261%), calcium (0.0296%), iodine (0.0339%), potassium (0.16%), lanthanum (0.0623%), magnesium (0.0466%), phosphorus (0.0459%), rhenium (0.0891%), silicon (0.0211%) and sulfur (0.0172%) (Fig. 2d).

### Cytotoxicity evaluation

The cytotoxic effects of nanoparticles (CS NPs, and C.CS NPs), herbal extracts (Iv, Sc, and Gg), herbal extract-loaded CS NPs (Iv-CS, Sc-CS, and Gg-CS), and herbal extract-loaded C.CS NPs (Iv-C.CS, Sc-C.CS, and Gg-C.CS) on HEK-293 and WI-38 cell lines were assessed using the WST-1 cell proliferation assay. At a low concentration of 100 µg/ml, herbal extracts, nanoparticles, and their loaded forms were non-toxic to both cell lines (Fig. 2e). Additionally, dexamethasone exhibited no toxicity up to a concentration of 40 µg/ml.

### Immunological parameters

Immunosuppressed rats showed a significant decrease in TLC, IFN_ϒ,_ C3, and C4 throughout the experimental period; however, in contrast, TNF level was elevated compared with the control group.

**Fig. 3:**
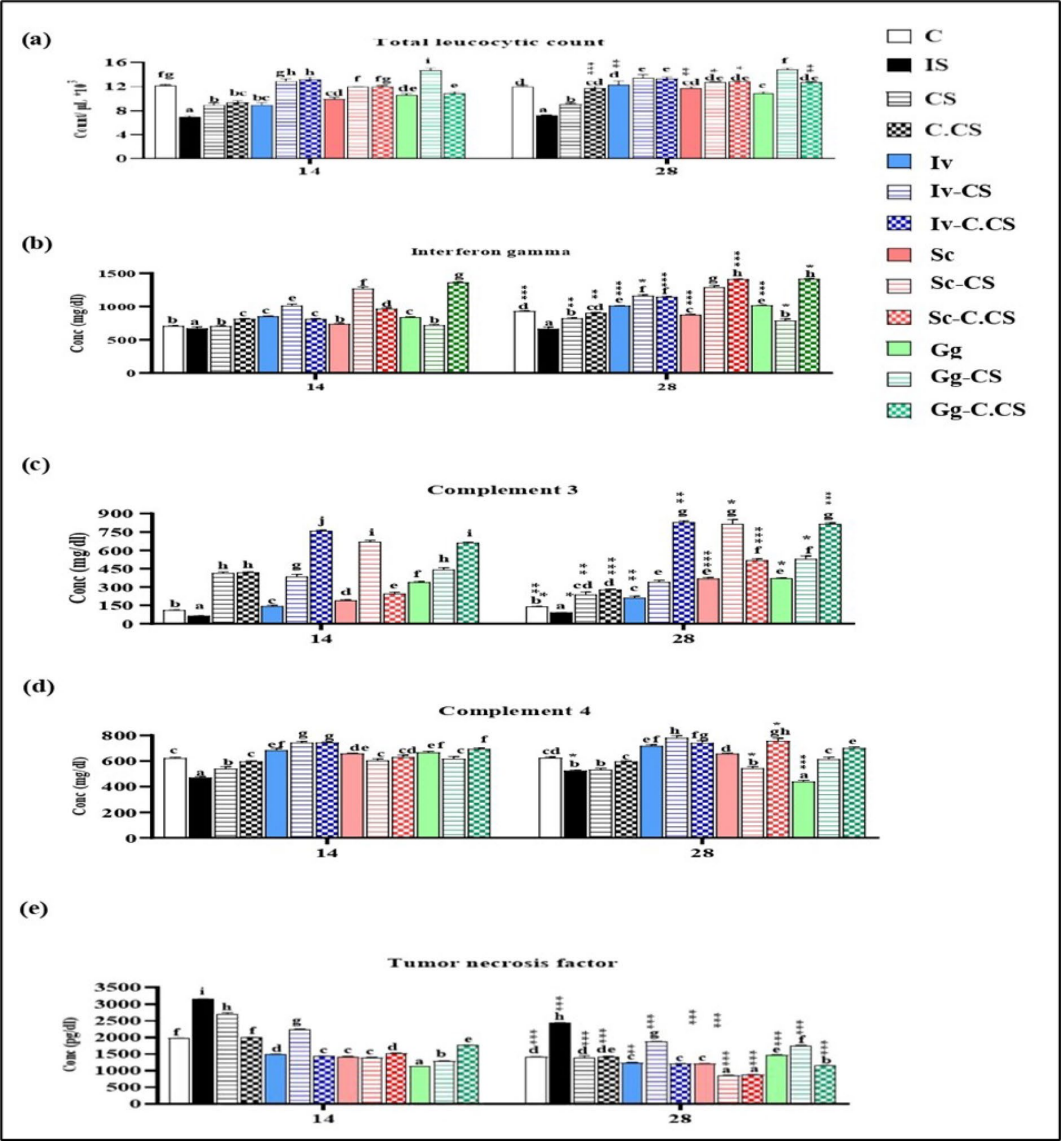
graphs display immunological parameters measured on days 14 and 28, including (**a**) Total leucocytic count, (**b**) Complement 3, (**c**) Complement 4, (**d**) Interferon gamma, and (**e**) Tumor necrosis factor. Data are shown as mean ± SE (*n* = 8) and analyzed with one-way ANOVA and Duncan test for group comparisons. A paired sample t-test was used to evaluate results within the same group between the two days. Significant differences are indicated by lowercase letters for day 14 and uppercase letters for day 28, along with symbols denoting differences between days: * for *P* ≤ 0.033, ** for *P* ≤ 0.002, and *** for *P* ≤ 0.001.

TLC was significantly enhanced in all treated groups compared to the IS one. The CS loaded groups exhibited higher values than those treated with CS NPs and herbal extracts. Additionally, the C.CS loaded groups had higher TLC values than the C.CS NPs and herbal extract groups, except for Gg-C.CS. When comparing the C.CS loaded groups to those of CS, Gg-CS showed higher TLC results than Gg-C.CS. The same outcomes were observed at day 28, except for Sc-CS and Sc-C.CS, which demonstrated similar results compared to the saussurea extract. Collectively, when comparing results of 14 and 28 days, we found that TLC was significantly higher at day 28 than at day 14 in C.CS, Iv, Sc, Sc-CS, Sc-C.CS and Gg-C.CS groups (Fig. 3a).

Herbal extracts, loaded herbal extracts, and nanoparticles exhibited marked elevation in IFN_ϒ_ in contrast to IS rats at day 14. IFNϒ level was higher in CS loaded herbs than in herbal extract and CS NPs, except in Gg-CS. C.CS loaded herbal extract exhibited a higher level than C.CS and herbal extract, except in Iv-C.CS. When comparing CS loaded herbs to those of C.CS, we found that C.CS - loaded herbs illustrated higher values than those of CS in Gg-C.CS. On day 28, IFN-γ levels significantly increased in all groups compared to the IS group, except for the CS, C.CS, Sc, and Gg-CS groups. C.CS - loaded herbal extract demonstrated the highest level among all other treated groups, except in Iv-C.CS that didn’t show any change when compared to Iv-CS. IFNϒ levels were generally higher on day 28 compared to day 14 across all except for of the Sc-CS group (Fig. 3d).

C3 was significantly elevated in all treated groups in comparison to IS at day 14. CS and C.CS Loaded herbal extracts groups exhibited a higher elevation in C3 level than herbal extracts only. CS loaded extracts exhibited a higher increase in C3 level compared with those in CS NPs group at day 14 except in Iv-CS, C.CS Loaded herbal extracts demonstrated a higher increase than C.CS NPs at day 14, except in Sc-C.CS. In comparing CS and C.CS loaded groups, Iv-C.CS, Gg-C.CS and Sc-CS were higher. The same results were obtained at day 28 except in Iv-CS and Sc-C.CS. Collectively, the C3 levels had notably increased on day 28 compared to day 14 across all groups, except for CS, C.CS, and Iv-CS (Fig. 3b).

C4 was significantly elevated in all groups in relation to IS one at day 14. CS loaded groups illustrated higher values than CS and herbal extracts, except in Iv-CS, C.CS loaded herbal extracts exhibited better results than C.CS except in Sc-C.CS also they showed better results than herbal extracts, except in Iv-C.CS. In contrast, the results of CS loaded groups compared with those of C.CS, it was found that C.CS - loaded herbs showed higher results in Gg-C.CS. C4 levels at day 28 were significantly elevated in all groups compared to IS, except for Gg group.C4 values were raised among the CS loaded groups, except for Sc-CS. Similarly, C.CS - loading herbal extracts demonstrated better results than in C.CS and herbal extracts, except for Iv-C.CS. Comparing the results of CS-loaded groups to those of C.CS, it was observed that C.CS - loaded herbal extracts had higher values, except for Iv-C.CS. In the Sc-C.CS group, C4 levels were significantly elevated on day 28 compared to day 14. (Fig. 3c)

Throughout the experimental period, all treated groups showed a significant reduction in TNF levels compared to the IS group. On day 14, CS-loaded herbal extracts groups exhibited lower results when compared to CS NPs. TNF level was lower in C.CS loaded herbal extracts groups than C.CS NPs, Iv-C.CS showed lower results than those of CS. On day 28, C.CS-loaded herbal extracts illustrated lower results than C.CS NPs and herbal extracts except in Iv-C.CS, which didn’t show any significant difference with illicium extract, in addition, C.CS loaded groups showed lower results than those of CS. However, Iv-CS and Gg-CS showed higher results than CS NPs. Overall, TNF levels were lower on day 28 compared to day 14 in all groups, except for the Gg and Gg-CS groups. (Fig. 3e).

### Biochemical parameters

The activity of ALT was significantly elevated in the immunosuppressed group on day 28 in comparison to the control group. However, it was significantly lowered in rats that received CS, Iv, Iv-C.CS, Sc-CS, Sc-C.CS, Gg and Gg-CS at day 28 of the experiment when compared to IS one (Table 2). When comparing treated groups with control one, ALT activity was significantly lowered in Iv but significantly increased in Gg-C.CS on day 14. Moreover, on day 28, it was markedly elevated in Sc, Gg-C.CS.

AST activity was significantly lowered in rats given Iv, Sc-CS, Sc-C.CS at day 14 but in Iv-CS, Sc, Gg-C.CS at day 28 and in Iv-C.CS during the experiment in comparison to immunosuppressed rats. Moreover, AST activity was significantly lowered in rats given Iv-C.CS, Sc-CS, Sc-C.CS at day 14 also lowered in Iv-CS, Iv-C.CS, and Gg-C.CS at day 28 in comparison to control rats.

Urea wasn’t significantly changed in all groups except in Sc at day 14 but, it was significantly reduced in all herbs, even extract or loaded and nanoparticles, when compared to the control one at day 28. Creatinine wasn’t significantly changed in all groups except in Sc-C.CS and Gg-C.CS in comparison to immunosuppressed, which decreased at day 14 (Table 2).

Tp didn’t change in IS, Iv, Iv-CS, Sc-CS, and Sc-C.CS however, it was significantly enhanced in CS, C.CS, Iv-C.CS, Sc, Gg, Gg-CS and Gg-C.CS at day 14, in contrast to control rats. On day 28 it didn’t alter in IS, Iv-CS, Iv-C.CS, Sc-C.CS and Gg-CS but it was significantly elevated in CS, C.CS, Iv, Sc-CS, Gg, Gg-C.CS in comparison to the control one. Alb was significantly increased in all treated groups, except in CS and C.CS, which didn’t show any significant difference when compared to control at day 14. The same results were obtained on day 28, except in CS. Glo wasn’t markedly altered in all groups except CS and C.CS which elevated at day 14, in contrast to control one. The same result was obtained at day 28, except CS, which didn’t show any significant difference when compared to the control one. Alb/Glo ratio wasn’t significantly changed in all treated groups, except in Sc-CS, which was elevated at day 14.

compared with that in the control group, but no significant differences were observed among the treated groups on day 28 (Table 3).

**Table 2:** demonstrates various biochemical parameters at both day 14 and day 28, including alanine aminotransferase (ALT), Aspartate aminotransferase **(**AST), Urea, and Creatinine.

Parameters groups	ALT (U/L)		AST (U/L)		Urea (mg/dl)		Creatinine (mg/dl)	
	14 days	28 days	14 days	28 days	14 days	28 days	14 days	28 days
**C**	10.42 ± 0.46 ^**bcd**^	10 ± 1.02 ^**AB**^	16.16 ± 1.32^**cde**^	18.99 ± 0.70 ^**BCD**^	15.11 ± 1.29^**abc**^	26.02 ± 0.93^**D**^	2.80 ± 0.51^**abc**^	3.31 ± 0.42^**AB**^
**IS**	7.43 ± 0.76 ^**a**^	13.93 ± 1.01^**C**^	17.87 ± 1.25^**def**^	19.43 ± 0.67^**CDE**^	12.36 ± 1.08^**a**^	23.11 ± 2.04^**CD**^	3.19 ± 0.49^**cd**^	3.03 ± 0.35^**AB**^
**CS**	11.04 ± 0.70 ^**cd**^	8.44 ± 0.51 ^**A**^	18.53 ± 0.74^**def**^	20.06 ± 0.45^**DEF**^	16.67 ± 0.75^**bcd**^	19.55 ± 0.57^**ABC**^	2.17 ± 0.24^**abc**^	2.56 ± 0.10^**A**^
**C.CS**	12.08 ± 0.58^**d**^	11.93 ± 0.48 ^**BC**^	18.99 ± 0.71^**ef**^	22.32 ± 0.63 ^**F**^	17.74 ± 0.77^**cd**^	20.11 ± 0.87^**BC**^	2.79 ± 0.23^**abc**^	3.06 ± 0.34^**AB**^
**Iv**	7.47 ± 0.67 ^**a**^	7.49 ± 1.03 ^**A**^	14.63 ± 1.43^**abc**^	17.54 ± 0.77^**BCD**^	15.83 ± 1.38^**abc**^	18.23 ± 0.95^**AB**^	2.27 ± 0.45^**abc**^	2.52 ± 0.38^**A**^
**Iv-CS**	8.99 ± 0.52 ^**abc**^	11.69 ± 1.12 ^**BC**^	17.59 ± 0.41^**def**^	15.83 ± 0.83^**A**^	17.28 ± 0.82^**cd**^	17.01 ± 0.99^**AB**^	17.28 ± 0.82^**bc**^	4.06 ± 0.34^**B**^
**Iv-C.CS**	10.09 ± 0.81 ^**bcd**^	8.60 ± 0.53 ^**A**^	11.77 ± 1.27 ^**a**^	15.98 ± 0.99^**A**^	15.49 ± 1.00 ^**abc**^	15.81 ± 1.30^**A**^	4.19 ± 0.34^**d**^	3.69 ± 0.39^A**B**^
**Sc**	12.33 ± 0.50 ^**d**^	12.71 ± 0.69 ^**C**^	15.26 ± 0.64^**bcd**^	16.16 ± 0.74^**AB**^	20.32 ± 2.39^**d**^	21.09 ± 2.08^**BC**^	2.38 ± 0.26^**abc**^	3.04 ± 0.32^**AB**^
**Sc-CS**	7.96 ± 0.39 ^**ab**^	7.75 ± 0.19^**A**^	13.82 ± 0.72^**ab**^	16.59 ± 0.94^**ABC**^	17.13 ± 0.72^**bcd**^	18.13 ± 0.49^**AB**^	2.17 ± 0.40^**abc**^	3.33 ± 0.18^**AB**^
**Sc-C.CS**	8.41 ± 0.55 ^**abc**^	10.03 ± 1.10 ^**AB**^	11.87 ± 1.50^**a**^	17.11 ± 1.11 ^**BC**^	18.29 ±1.35 **cd**	17.67 ± 1.46^**AB**^	1.75 0.20 ± ^**ab**^	3.35 0.52 ± ^**AB**^
**Gg**	10 ± 1.08 ^**bcd**^	9.84 ± 0.87^**AB**^	18.05 ± 1.14^**def**^	18.57± 1.37^**BCD**^	16.88 ± 1.46**bcd**	18.08± 1.54^**AB**^	2.33± 0.46^**abc**^	3.43± 0.52^**AB**^
**Gg-CS**	8.88 ± 0.64 ^**abc**^	9.93 ± 0.85^**AB**^	20.03 ± 0.47 ^**f**^	22.08 ± 0.92^**EF**^	15.94 ± 0.67**abc**	17.96 1.08 ± ^**AB**^	2.71 ± 0.40 ^**abc**^	4.03 ± 0.22 ^**B**^
**Gg-C.CS**	15.21 ± 1.92 ^**e**^	13.1 ± 0.96 ^**C**^	15.65 ± 1.27 ^**cde**^	15.73 ± 1.06 ^**A**^	13.21 ± 0.79 ^**ab**^	18.41 ± 1.22 ^**AB**^	1.70 ± 0.30 ^**a**^	3.57 ± 0.51 ^**AB**^

Values have the different superscripts are significantly different at P≤0.05 according to one-way ANOVA followed by the Duncan test

**Table 3:** Biochemical parameters at day 14 and day 28, including total protein, albumin, globulin, and albumin - globulin ratio.

Parameters groups	Total protein (g/dl)		Albumin (g/dl)		Globulin (g/dl)		Albumin/globulin ratio	
	14 days	28 days	14 days	28 days	14 days	28 days	14 days	28 days
**C**	7.17 ± 0.38 ^**a**^	7.86 ± 0.20 ^**AB**^	2.93 ± 0.20 ^**a**^	3.55 ± 0.20 ^**A**^	4.24 ± 0.45 **abc**	4.31 ± 0.39 ^**B**^	0.73 ± 0.11 ^**ab**^	0.89 ± 0.17 ^**AB**^
**IS**	7.64 ± 0.23 ^**ab**^	8.39 ± 0.16 ^**BCD**^	3.76 ± 0.21 ^**bcd**^	4.49 ± 0.31 ^**BCD**^	3.88 ± 0.25 ^**abc**^	3.90 ± 0.27 ^**AB**^	1.00 ± 0.12 ^**bc**^	1.20 ± 0.16 ^**B**^
**CS**	8.83 ± 0.28 ^**c**^	9.06 ± 0.20 ^**DE**^	3.23 ± 0.22 ^**ab**^	4.64 ± 0.35 ^**CD**^	5.60 ± 0.18 ^**e**^	4.42 ± 0.17 ^**B**^	0.58 ± 0.04 ^**a**^	1.07 ± 0.12 ^**AB**^
**C.CS**	8.81 ± 0.15 ^**c**^	9.33 ± 0.18 ^**E**^	3.45 ± 0.15 ^**abc**^	3.84 ± 0.11 ^**ABC**^	5.36 ± 0.15 ^**de**^	5.49 ± 0.21 ^**C**^	0.65 ± 0.04 ^**a**^	0.71 ±0.04 ^**A**^
**Iv**	7.61 ± 0.15 ^**ab**^	8.55 ± 0.24 ^**CDE**^	3.90 ± 0.20 ^**bcd**^	4.22 ± 0.13^**BCD**^	3.71 ± 0.15 ^**ab**^	4.33 ± 0.15 ^**B**^	1.06 ± 0.09 ^**bc**^	0.98 ± 0.03 ^**AB**^
**Iv-CS**	7.94 ± 0.20 ^**ab**^	8.18 ± 0.21 ^**BC**^	4.06 ± 0.19 ^**cd**^	3.76 ± 0.27 ^**AB**^	3.86 ± 0.08 ^**ab**^	4.43 ± 0.33 ^**B**^	1.07 ± 0.17 ^**bc**^	0.90 ± 0.15 ^**AB**^
**Iv-C.CS**	8.39 ± 0.29 ^**bc**^	8.43 ± 0.15 ^**BCD**^	3.88 ± 0.30 ^**bcd**^	4.60 ± 0.34 ^**BCD**^	4.51 ± 0.14 ^**bcd**^	3.83 ± 0.4 ^**AB**^	0.91 ± 0.40 ^**ab**^	1.26 ± 0.18 ^**B**^
**Sc**	8.30 ± 0.33 ^**bc**^	7.41 ± 0.15 ^**A**^	4.12 ± 0.26 ^**cd**^	4.07 ± 0.21 ^**BCD**^	4.18 ± 0.30 ^**abc**^	3.35 ± 0.32 ^**A**^	1.018 ± 0.13 ^**bc**^	1.29 ± 0.19 ^**B**^
**Sc-CS**	7.87 ± 0.13 ^**ab**^	8.88 ± 0.20 ^**CDE**^	4.43 ± 0.11 ^**d**^	4.12 ± 0.20 ^**BCD**^	3.44 ±0.04 **a**	4.78 ± 0.08 ^**BC**^	1.29 ±0.03 ^**c**^	0.86 ± 0.05 ^**AB**^
**Sc-C.CS**	7.68 ± 0.19 ^**ab**^	8.44 ± 0.42 ^**BCD**^	3.66 ±0.21 ^**bc**^	3.93 ± 0.27 ^**BCD**^	4.02 ±0.11**abc**	4.51 ± 0.30 ^**B**^	0.92 ± 0.07 ^**ab**^	0.88 ±0.073 ^**AB**^
**Gg**	8.32 ± 0.39 ^**bc**^	8.95 ±0.32 ^**CDE**^	3.77 ±0.35 ^**bcd**^	4.76 ±0.16 ^**D**^	4.55 ±0.39 **bcd**	4.19 ±0.22 ^**AB**^	0.86 ±0.13 ^**ab**^	1.15 ±0.06 ^**AB**^
**Gg-CS**	8.26 ± 0.37 ^**bc**^	8.36 ± 0.37 ^**BCD**^	4.02 ± 0.20 ^**cd**^	4.41 ± 0.08 ^**BCD**^	4.23 ±0.53**abc**	3.95 ± 0.41^**AB**^	1.04 ± 0.20 ^**bc**^	1.17 ± 0.15 ^**B**^
**Gg-C.CS**	8.49 ± 0.08 ^**bc**^	8.99 ± 0.26 ^**CDE**^	3.66 ± 0.12 ^**bc**^	4.39 ± 0.50 ^**BCD**^	4.83 ± 0.15 ^**cde**^	4.60 ± 0.45 ^**BC**^	0.76 ±0.05 ^**ab**^	1.03 ± 0.21 ^**AB**^

The data are expressed as mean ± SE (n = 8) and analyzed using one-way ANOVA with Duncan post-hoc test for comparisons on days 14 and 28. Significant differences at P≤ 0.05 are indicated by different letters, with lowercase for day 14 and uppercase for day 28. A paired sample t-test was also performed to compare results within the same group across the two days, marked by asterisks (*) for significant differences

### Histopathological findings

Histopathological examination of the spleen in the control group revealed a white pulp formed of peri-artiolar lymphoid sheath, lymphoid follicles, and marginal zone, and a red pulp formed of venous sinuses and splenic cords (Fig. 4.1a). In the IS group, the white pulp was atrophied with expansion of the red pulp. The lymphocytes were depleted from the peri-artiolar lymphoid sheath with the ill distinction of lymphoid follicles, poorly formed germinal centers, and disappearance of marginal zone (Fig. 4.1b). In CS group, the white pulp, including the peri-artiolar lymphoid sheath, lymphoid follicles, and marginal zone, was repopulated by lymphocytes (Fig. 4.1c). The white pulp was moderately repopulated by lymphocytes in C.CS (Fig. 4.1d), mildly populated in Iv group (Fig. 4.1e), moderately populated in Iv-CS group (Fig. 4.1f), and Iv-C.CS group (Fig. 4.1g), highly populated in Sc group (Fig. 4.1h), and Sc-CS group (Fig. 4.1i), moderately populated in Sc-C.CS group (Fig. 4.1j), Gg group (Fig. 4.1k), Gg-CS group (Fig. 4.1l), and Gg-C.CS group (Fig. 4.1m).

Microscopy of liver and kidney in different treated groups revealed normal histological structure (Fig. 5.1, Fig. 5.2).

**Fig. 4:**
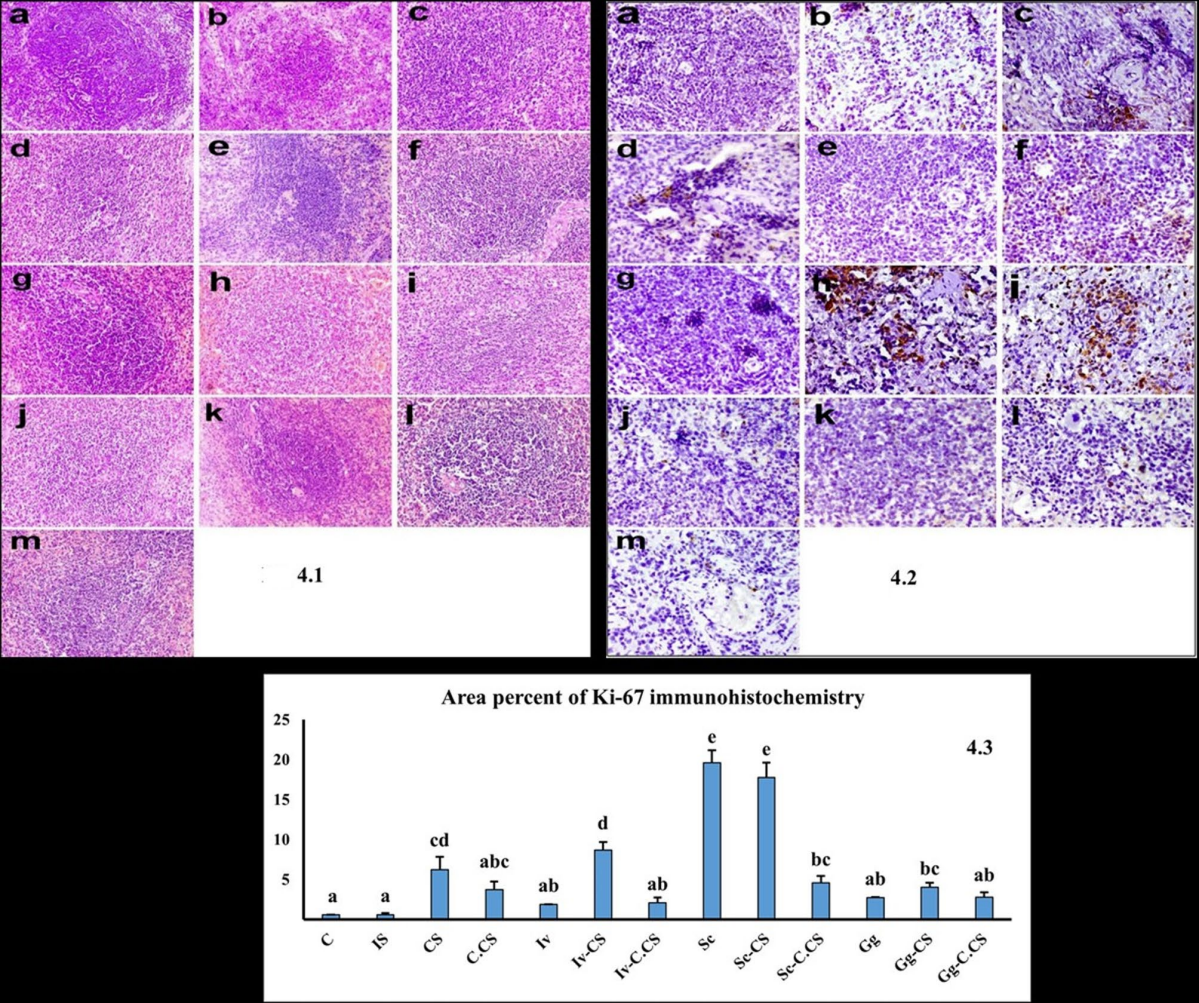
Fig 4.1 Spleen tissue of rats across different groups: (**a**) In the control group, the white pulp is clearly defined with its three compartments: periarteriolar lymphoid sheath, lymphoid follicles, and marginal zone. (**b**) The IS group shows white pulp atrophy and lymphocyte depletion. (**c**) The CS group exhibits repopulation of the white pulp (**d**) C.CS group, (**e**) Iv group, (**f**) Iv-CS group, (**g**) Iv-C.CS group, (**h**) Sc group, (**i**) Sc-CS group, (**j**) Sc- C.CS group, (**k**) Gg group, (**l**) Gg-CS group, (**m**) Gg-C.CS group. (Hematoxylin and eosin stain X 200).Fig 4.2 Ki-67 immunohistochemistry in splenic tissue of rats in different groups. (**a**) Weak expression of Ki-67 in the white pulp of control group and (**b**) IS group. (**c**) the expression of Ki-67 was observed in the lymphocytes populating the white pulp in CS group, (**d**) C.CS group, (**e**) Iv group, (**f**) Iv-CS group, (**g**) Iv-C.CS group, (**h**) Sc group, (**i**) Sc-CS group, (**J**) Sc-C.CS group, (**k**) Gg group, (**l**) Gg-CS, (**m**) Gg-C.CS group. (Immunoperoxidase X400).Fig 4.3Ki-67 immunohistochemistry area percentages in splenic tissue of different rat groups. The columns indicate mean area percentages, with thin lines representing standard errors. Columns with different lowercase letters denote significant differences at p < 0.05.

**Fig. 5:**
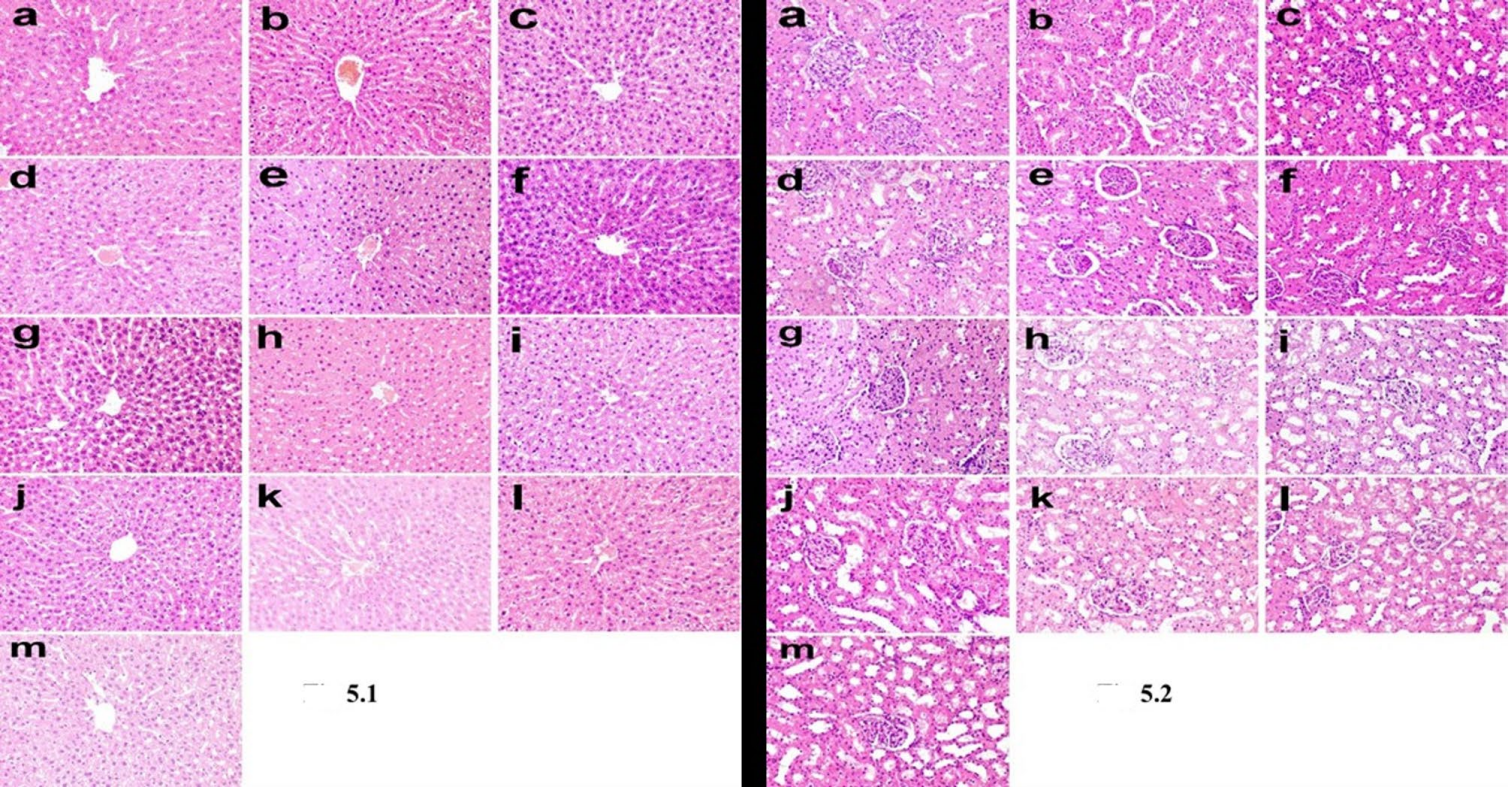
Fig 5.1The liver tissues of rats across various treatment groups exhibit normal histological features. (**a**) Control group, (**b**) IS group, (**c**) CS group, (**d**) C.CS group, (**e**) Iv group, (**f**) Iv-CS group, (**g**) Iv-C.CS group, (**h**) Sc group, (**i**) Sc-CS group, (**j**) Sc- C.CS group, (**k**) Gg group, (**l**) Gg-CS group, (**m**) Gg-C.CS group. (Hematoxylin and eosin stain X 200). Fig 5.2 The kidneys of rats in the various treatment groups display normal histological architecture. (**a**) Control group, (**b**) IS group, (**c**) CS group, (**d**) C.CS group, (**e**) Iv group, (**f**) Iv-CS group, (**g**) Iv-C.CS group, (**h**) Sc group, (**i**) Sc-CS group, (**j**) Sc- C.CS group, (**k**) Gg group, (**l**) Gg-CS group, (**m**) Gg-C.CS group. (Hematoxylin and eosin stain X 200).

### Ki-67 immunohistochemistry results

Minimal Ki-67 expression was observed in the lymphocytes of the white pulp in both the control and IS groups (Fig. 4.2a, b). Conversely, in all treated groups, Ki-67 expression varied among the lymphocytes repopulating the white pulp (Fig. 4.2c-m). Notably, the CS, Iv-CS, Sc, Sc-CS, Sc-C.CS, and Gg-CS groups showed a significant increase in the area percentage of Ki-67 expression compared to the control group (Fig. 4.3). The most substantial increase in Ki-67 expression was observed in the Sc and Sc-CS groups.

## Discussion

Glucocorticoids (GCs) are potent anti-inflammatory and immunosuppressive drugs that exert their effects by interacting with the intracellular glucocorticoid receptor (GR), which is found in both innate and adaptive immune cells. Dexamethasone therapies, which are a type of GC, can impact various immune cells, including both lymphoid and myeloid cells, either by reducing their numbers or by decreasing their immune function, likely through the GR signaling pathway. This finding could increase the clinical usefulness of dexamethasone in inducing temporary immune suppression. In addition, Dexamethasone works by suppressing the production of various cytokines, including interleukin-1 (IL-1), interleukin-2 (IL-2), and interleukin-6 (IL-6), which regulate the activity of immune cells^39^.

In the current study, intraperitoneal injection of dexamethasone induces immunosuppression through decreasing TLC, IFN_ϒ,_ C3, and C4 levels with increasing TNF level. These results were found to be in accordance with^40^ who declared that dexamethasone injection can lead to a decrease in TLC level, as well as a decrease in other immunological parameters. Also, dexamethasone can significantly reduce the production of IFN gamma by T cells and natural killer (NK) cells^41^. Complement system proteins play a critical role in the clearance of pathogens and are involved in the immune system’s response to foreign invaders^42^. found that corticosteroids can decrease the levels of both C3 and C4. Moreover^43^, stated that treatment with dexamethasone increases the production of TNF by immune cells, as it can increase the expression of TNF receptors on immune cells. These findings were confirmed by the histopathological picture of an immunosuppressed spleen in which the white pulp was atrophied with low expression of Ki-67. This is in accordance with the results obtained by who showed that treatment with dexamethasone had a negative impact on the white pulp of the spleen, resulting in impaired differentiation of tissue compartments.

Phytoconstituents or phytochemical ingredients are various chemical compounds found in herbs including flavonoids, quinine, terpenoids, and others. They play a crucial role in carrying out specific biological functions that contribute to therapeutic properties of herbs, such as anti-carcinogenic, anti-mutagenic, anti-inflammatory, antioxidant effects, as well as immunomodulatory actions^44^. In the present study, antioxidant activity and phenolic content for illicium (93.74% and 23.04 mg/g respectively) were in agreement with^45^ who found that these contents are about 95.67% and 28.59 mg/g respectively. Gg antioxidant activity is about 87.92% which coincided with^46^ who stated that Gg total antioxidant activity is about 88.7%. Sc phenolic was 14.74 mg/g which agreed with that obtained by Singh et al. 2018 (12.45 to 75.02 mg/g). Collectively, Iv exhibited the highest antioxidant contents, phenolic, and flavonoids (93.74%, 23.04 mg GAE/g and 19.76 mg QE/g respectively) compared to Gg and Sc.

Throughout the experiment, rats given oral doses of various herbal extracts exhibited a stronger immune response compared to the immunosuppressed group. This was evidenced by higher levels of TLC, IFN-γ, C3, and C4 along with a reduction in TNF-α levels. All herbs used in the present study stimulate and improve immunity; this may be due to their high flavonoid content, which is considered a potential factor contributing to the observed enhancement in immune response. Furthermore, the beneficial effects observed may be due to the essential elements found in these extracts, such as potassium, sodium, and calcium, which are crucial for immune system support^47^ reported that the presence of sodium can lead to an increase in proinflammatory macrophage and T cell activities, while concurrently inhibiting anti-inflammatory immune responses^48^. stated that potassium flux also contributes to critical immunological and antimicrobial processes, such as cytokine production and inflammasome activation^49^. showed that Calcium signaling plays a crucial role in the activation and proliferation of T cells when they are stimulated by antigens. The immunomodulatory results were supported by^50^, who stated that *Illicium verum* has been shown to possess immunostimulant properties. The active components responsible for these effects are believed to be the polyphenolic compounds present in the herbs, including polysaccharides, alkaloids, or flavonoids. Also, it can stimulate the immune system by increasing phagocytic activity, activation of the complement system, and increasing respiratory burst activity^51^. reported that *Saussurea costus* appears to have significant immunostimulant properties by increasing the proliferation of leukocytes and lymphoid tissue. It stimulates phagocytosis by macrophage activation, increases the polymorphonuclear cells, antibody titer value, and responsiveness of macrophages, T and B lymphocytes^52^. demonstrated the immunostimulant effect of *Glycyrrhiza glabra* through an increase in total leucocytic count, phagocytic and lysozyme activity, enhancing lymphocyte and monocyte percentages.

Current study clarified that administration of nanoparticles orally improves immune response by increasing TLC, IFN-γ, C3, C4 levels, and decreasing TNF-α level. This was due to the immunostimulant effect of chitosan nanoparticles and their modification, and carboxy methyl chitosan exhibited better results than chitosan during the experimental period. These results were supported by^53^ who reported that carboxy methyl chitosan increases the expression of cytokines (IL-6 andIL-1β) and the secretion of four immunity-related membrane proteins (MHC-II, CD80, CD86, and CD11c)^54^. investigated the chitosan immunostimulant effect, which may be via promoting the functions of polymorphonuclear leucocytes, cytokines, and macrophages.

The chitosan (CS) or carboxymethyl chitosan (C.CS) loaded herbal extracts demonstrated a more pronounced immunostimulatory effect compared to the herbal extract alone. This was evidenced by significantly higher values of total leukocyte count (TLC) and key immunological markers such as complement components C3 and C4, and interferon-gamma (IFN-γ), along with a notable reduction in tumor necrosis factor-alpha (TNF-α) levels. These findings are supported by^23^, who reported that chitosan nanoparticles (CS NPs) and their conjugates with herbal extracts significantly enhanced TLC, C3, C4, and IFN-γ levels, while reducing TNF-α compared to the use of herbal extracts alone. Furthermore, the current results align with the observations of^55^, who highlighted several advantages of nanoparticle-loaded herbal extracts. These include improved ability to cross biological barriers, enhanced bioavailability of poorly water-soluble phytochemicals, and targeted delivery to specific organs. Additionally, such nanoparticles protect phytochemicals from degradation due to biological factors (e.g., pH) and environmental conditions (e.g., temperature, light, and humidity), while also enabling controlled release.

Carboxy chitosan loaded herbal extracts groups illustrated better immune response than those of chitosan alone. This may be due to C.CS having several advantages over chitosan nanoparticles. C.CS is a water-soluble derivative of chitosan, and it has better solubility in aqueous solutions when compared to chitosan. It possesses lower toxicity compared to chitosan, primarily because of its enhanced water solubility and reduced capacity to interact with cell membranes. This attribute makes it a safer material for biomedical utilization^56^. C.CS displays increased stability as compared to chitosan, owing to its charge density that prevents particle aggregation and enhances drug delivery and shelf-life stability. Furthermore, C.CS nanoparticles exhibit superior mucoadhesive properties than chitosan, owing to the presence of carboxymethyl groups, which improve adhesion to mucosal surfaces, thereby enhancing drug absorption and retention. C.CS nanoparticles also offer a more regulated drug release profile than chitosan, owing to their superior stability and solubility properties, which enable sustained drug release. This attribute can lower the frequency of dosing and improve patient compliance^57^.

In the present study, it was observed that the utilization of both extracted and loaded herbs, as well as nanoparticles, did not have any adverse effects on liver and kidney functions. This outcome can be credited to the hepatoprotective and nephroprotective properties of I*llicium verum*^58,59^, *Saussurea costus*^60^, and *G. glabra*^61^. These properties can be attributed to the presence of various components, such as polyphenolic compounds, which have the potential to protect cells by enhancing the activity of antioxidant enzymes like CAT, SOD, and glutathione peroxidase, as well as increasing radical scavenging activity to mitigate lipid peroxidation. This can improve liver function markers and safeguard renal tissue from oxidative damage.

## Conclusion

The administration of dexamethasone at a dose of 20 mg/kg effectively induced immunosuppression in rats, as evidenced by significant alterations in key immunological biomarkers. Therapeutic intervention using extracts of Illicium verum, Saussurea costus, and Glycyrrhiza glabra at 200 mg/kg demonstrated a notable capacity to counteract these immunosuppressive effects. Furthermore, the incorporation of these herbal extracts into chitosan and carboxy chitosan (C.Cs) nanoparticle delivery systems markedly enhanced their immunostimulatory efficacy, even at a reduced dose of 100 mg/kg. Among the formulations tested, the C.Cs-based nanoparticles exhibited superior performance, likely due to their improved physicochemical properties, including enhanced stability, solubility, and mucoadhesiveness. These findings highlight the promising potential of nanoparticle-mediated delivery of herbal bioactives as a strategic approach to immunomodulation. The enhanced immune responses observed in the C.Cs-loaded groups underscore the advantages of this delivery system in optimizing therapeutic outcomes and overcoming limitations commonly associated with conventional herbal administration. While the present study primarily focused on immunostimulatory effects, the results pave the way for broader biomedical applications of these compounds, particularly in the context of immune-compromised conditions. From a clinical perspective, this approach may offer supportive therapy for individuals with weakened immune systems, such as patients undergoing chemotherapy, recovering from organ transplantation, or suffering from chronic infections. The use of biocompatible, naturally derived nanoparticles also presents an opportunity to reduce therapeutic dosages and improve patient compliance. On an industrial scale, the integration of herbal extracts with nanocarrier technologies opens new avenues for the development of immune-enhancing nutraceuticals and cosmeceuticals. This scalable and sustainable method offers a natural alternative to synthetic immunotherapies, with potential applications across the pharmaceutical and wellness sectors.

## Limitations

- The use of a single animal model (male Wistar rats) may limit generalizability across sexes and species.
- Molecular-level pathway validation (e.g., protein expression analyses such as Western blotting) was not included due to resource constraints.
- Long-term effects and dose–response relationships were beyond the scope of this study and warrant future investigation.
- Pharmacokinetic profiling and tissue distribution of nanoparticle-loaded formulations were not assessed but are planned for subsequent studies.

## Data Availability

Data is provided within the manuscript.
